# Assessing patient satisfaction with practitioner communication: patient-centered care, hospital environment and patient trust in the public hospitals

**DOI:** 10.3389/fmed.2025.1544498

**Published:** 2025-05-21

**Authors:** Arif Jameel, Noman Sahito, Wenjing Guo, Sania Khan

**Affiliations:** ^1^School of Business, Shandong Xiehe University, Jinan, China; ^2^Architecture and City Design Department, College of Design and Built Environment, King Fahd University of Petroleum and Minerals, Dhahran, Saudi Arabia; ^3^Interdisciplinary Research Center for Sustainable Energy System King Fahd University of Petroleum and Minerals, Dhahran, Saudi Arabia; ^4^Department of Human Resource Management, College of Business Administration, Prince Sattam Bin Abdulaziz University, Al Kharj, Saudi Arabia

**Keywords:** practitioner-patient-communication, patient satisfaction, patient trust, cleanliness, health communication, patient-centered care, Saudi Arabia

## Abstract

**Introduction:**

Patient satisfaction is increasingly an important concern in worldwide health policy. The purpose of this research is to identify the effect of practitioner-patient communication (PPC) and cleanliness on patient satisfaction (PS), as well as the mediating role of patient trust (PT).

**Methods:**

Data were obtained from 497 patients in public hospitals in Saudi Arabia. The data were analyzed using AMOS 25 and Structural equation modeling (SEM) techniques.

**Results:**

The results show that practitioner-patient communication and cleanliness positively impact patient satisfaction. Further, it reveals that patient trust mediates practitioner-patient communication, cleanliness, and patient satisfaction.

**Conclusion:**

The findings may benefit research and healthcare policy formulators since they examined the patients’ perspective of practitioner-patient communication, cleanliness, and satisfaction at Outpatient Departments of public hospitals. The study offers important insights for healthcare authorities to devise strategies to improve service delivery in public hospitals and ensure patient satisfaction.

## Introduction

Patient satisfaction (PS) and loyalty are essential elements of healthcare delivery, directly impacting the overall quality of service, patient retention, and the viability of healthcare facilities (1). The global healthcare business has transitioned from a provider-centered strategy to a patient-centered one that emphasizes patients’ needs, preferences, and satisfaction (2, 3). This paradigm change has been notably impactful in healthcare systems that seek to improve service quality, maximize patient outcomes, and cultivate enduring connections between patients and clinicians (4). In Saudi Arabia, the healthcare system has experienced significant transformations in the past few years, especially with the introduction of the Saudi Vision 2030 project, aimed at enhancing the quality of healthcare services and fostering patient happiness (5). Nonetheless, the elements influencing PS and loyalty in the Saudi setting, especially in the Riyadh, Damman, and Jeddah regions, are inadequately examined (6). PS denotes the degree to which individuals are pleased with the healthcare services (7). It is a multifaceted notion that includes several dimensions of healthcare delivery, such as the quality of treatment, communication between patients and providers, the responsiveness of healthcare personnel, and the general atmosphere of healthcare facilities (8). Studies have repeatedly demonstrated that pleased patients are more inclined to comply with treatment regimens, achieve superior health results, and cultivate confidence and loyalty toward their healthcare providers (9). Dissatisfaction with healthcare services can result in worse health outcomes, diminished healthcare system faith, and heightened provider change rates (10, 11). Patient satisfaction serves as a critical performance metric for healthcare institutions. It indicates the efficacy of service provision, the proficiency of healthcare personnel, and the organization’s capacity to fulfill patient requirements (12). Communication between practitioners and patients may be complex and multidimensional. A practitioner’s communication and interpersonal skills encompass gathering information for precise diagnosis, providing effective counseling, providing treatment instructions, and cultivating empathetic relationships with patients (1, 13). The essential clinical competencies necessary for medical practice aim to achieve optimal outcomes and patient satisfaction, which are critical for the efficient provision of healthcare (14). Effective practitioner-patient communication involves coaching patients on unhealthy or dangerous behaviors and is an essential skill that should be included in all medical consultations. Comprehending behavioral modifications and establishing a systematic treatment framework, incorporating the five A’s (assess, advise, agree, assist, and arrange) of patient counseling, are critical components of practitioner-patient communication (15). Effective communication between practitioner and patient functions as a motivator, incentive, source of confidence, and encouragement for the patient (16–18). Moreover, proficient practitioner-patient communication can aid patients in regulating their emotions, enhancing the comprehension of medical information, and enabling a more precise evaluation of their needs, perceptions, and expectations (19–21). Practitioner and patient agreement on the treatment’s nature and the need for follow-up significantly correlates with recovery (22, 23). Effective communication between patients and Practitioners enhances satisfaction with treatment and facilitates the exchange of essential information necessary for correct diagnosis, adherence to recommendations, and compliance with prescribed therapies (24). In Saudi Arabia, where the healthcare system is experiencing swift development, assessing and enhancing patient satisfaction has become a priority for public and commercial healthcare organizations (25, 26). PS in Saudi Arabia is affected by several cultural, societal, and organizational aspects that may vary from those in other areas (27). Consequently, there is a want for context-specific research that delineates the distinct aspects influencing patient satisfaction among Saudi patients. Patient loyalty or trust is intricately linked to contentment, although it explicitly denotes the probability that a patient would persist in utilizing the same healthcare practitioner or institution throughout time (28). Trust is essential for the sustained success of healthcare organizations, as retaining current patients is more cost-effective than acquiring new ones (29). Furthermore, devoted patients are more inclined to recommend others to the healthcare institution, enhancing the company’s brand and expanding its patient base (30). In healthcare environments, patient trust is frequently influenced by favorable experiences, confidence in healthcare personnel, and assurance over the quality of service (31). Trust is contingent upon the perceived value of healthcare services, encompassing both clinical outcomes and the total patient experience (32). In Saudi Arabia, where patients can utilize both public and private healthcare facilities, trust and loyalty may be affected by several variables, such as the availability of specialized treatments, the reputation of healthcare professionals, and cultural concerns (27). Research on patient trust as a mediator within Saudi healthcare environments is scarce, necessitating an investigation into the specific elements that influence patient trust among Saudi patients, especially in the Riyadh, Damman and Jeddah region. Numerous variables may affect PS and loyalty in Saudi Arabia, including cultural norms, religious beliefs, communication techniques, and the organizational structure of healthcare institutions (7). In Saudi Arabia, the cultural focus on privacy and modesty may influence patients’ expectations of healthcare services, especially with gender-segregated treatment and the presence of female healthcare personnel for female patients (33). PS and loyalty are essential elements of healthcare delivery, directly affecting the overall quality of care, patient retention, and the success of healthcare facilities (34–36). Numerous global researches have established a correlation between patient satisfaction, treatment adherence, and long-term loyalty, underscoring the significance of communication, service quality, and patient-provider relationships in cultivating patient loyalty (37–40). In Saudi Arabia, research about patient satisfaction is yet in its nascent stages. Although several studies have concentrated on clinical outcomes and organizational performance Mahfouz (41), few research has examined the particular elements affecting patient satisfaction and loyalty in the region, especially in the swiftly transforming healthcare environment under the Saudi Vision 2030 project (7). Current research highlights the significance of practitioner communication and empathy in influencing patient experiences. Rahman and Al-Borie (42), although empirical information about their impact on trust, especially in Outpatient departments of public hospital, remains scarce. This study aims to address this gap by analyzing the primary determinants of patient satisfaction and mediating role of trust among Saudi patients visiting healthcare institutions. This research tries to elucidate how many elements of the healthcare experience, including the frequency of healthcare visits, the kind of institution, and patient demographics, contribute to patient satisfaction and long-term loyalty. These results will not only guide healthcare practitioners in Saudi Arabia but also enhance the worldwide dialogue on optimizing patient experiences in emerging healthcare systems.

## Literature review and hypotheses development

### An overview of the Saudi healthcare system

Saudi Arabia is among the largest nations in West Asia, boasting a population of 34 million individuals. The healthcare system in the Kingdom has experienced substantial enhancements due to the government’s considerable expenditures in healthcare infrastructure, leading to improved access to healthcare services nationwide. Saudi Arabia’s robust healthcare system offers complimentary medical care to all citizens and residents. The government is accountable for delivering healthcare services and serves as the principal financier of the healthcare system (43). The Ministry of Health (MOH) oversees the regulation of the healthcare system and the provision of healthcare services nationwide. The healthcare system in the Kingdom comprises basic, secondary, and tertiary healthcare services. Primary healthcare services are delivered by primary healthcare centers (PHCs), which offer fundamental healthcare services, encompassing preventative care, health education, and screening services. Secondary healthcare services are delivered via hospitals and specialized centers, offering sophisticated medical services such as diagnostic evaluations, surgical interventions, and emergency treatment. Tertiary healthcare services are delivered by specialist hospitals that provide advanced medical care, including transplantation and oncology treatments (44, 45).

### General practitioner-communication and patient satisfaction

General practitioner-patient communication is critical since people usually choose their common view of medical services and their possible results (28, 46). They should create a good and cordial connection with the practitioner to evaluate medical services through improved communication (47, 48). A positive association between practitioner and patient is essential for ensuring that the patient follows the health advice they have acquired, which eventually enhances effective treatment and lowers expenditures (49, 50). This connection might also increase the patient’s confidence and desire to receive such health facilities. This repeated use can help improve the hospital’s reputation (51, 52). Several researchers have also discovered that a patient’s level of health directly affects their satisfaction levels (53–55), with patients who require deeper insights into medical treatment having a higher perception of the quality of the physician’s services, in addition to their general contentment. Mekoth (56) revealed that a practitioner’s checkup and communication abilities at outpatient facilities might impact patient perceptions. According to Hussain (57), five constructive underlying factors encompass hospital personnel proficiency and skills, medicinal efficacy, the atmosphere they establish and the support they provide, general perception, and service processes. The World Body has developed “sustainable development goals” (SDGs) that emphasize the significance of enhanced delivery of services by facilitators (58).

*H1*: There is a positive relation between general PPC and PS.

### Cleanliness and patient satisfaction

Cleanliness relates to sanitation in the service atmosphere. The reception desk’s clean environment, the sidewalk leading to the health center, the examination room, the sitting area, and the medical staff attire. Several studies have found the positive impact of cleanliness in, e.g., Maternity care patient rooms, technological laboratories, patient satisfaction with “diagnostic test rooms, blood banks,” wards, beds, ambulance services, and operating theaters (59–61). Awan (62) investigated the degree of cleanliness in the hospitality industry during the outbreak, concluding that cleanliness was a critical element influencing consumers’ confidence and pleasure. Cleanliness or hygienic aspects are viewed as physical characteristics of the service environment that promote satisfaction and trust within the emergency room and outpatient care (63). Early research has demonstrated the impact of cleanliness on patient contentment and satisfaction in medical-care providers, where the emphasis has been on “cleanliness” in hospitals, restrooms, and surroundings for patient care and satisfaction (64, 65). Tidy waiting rooms can impact patient satisfaction with medical services. Javed (66) discovered that cleanliness influences patient satisfaction in the emergency room. We propose, based on these indications, that:

*H2*: Cleanliness in hospitals is positively related to PS.

### Mediating effect of patient trust

Trust is a vital aspect of practitioner-patient communication, and the PT in the practitioner is the foremost significant facet (67). Trust reduces clients’ behavioral risks, which leads to consumer satisfaction. Once trust is formed, there is no reason to create a cost control system, enhancing longer-run association advantages (68). Trust is the anticipation that a person or a team will sincerely attempt to behave in line with promises (both express and indirect), to be truthful, and not to exploit people even if the occasion arises (69). This concept of trust emphasizes positive notions of trust, a readiness to tolerate risk, or both (70, 71). Patients’ trust increases their self-belief that healthcare practitioners have stronger intention and an interpersonal connection, particularly in the practitioner-patient relation (PPR) Mechanic and Schlesinger (72), which is at the center of interactive communication in quality medical care (73). Establishing a method to govern a physician’s contact with patients, known as practitioner-patient communication (PPC), is critical to increasing trust in health personnel and decreasing patients’ risk perception. PPC includes communication abilities, gentle values, and professionalism (74). Patients are much more inclined to trust healthcare practitioners when they feel welcomed and consider them responsive and sincere (75). According to the conceptual model proposed by Bustamante (76), a gain in trust from sharing information significantly influences patients’ perceptions of healthcare practitioners. Interventions to enhance doctors’ communication skills have also boosted trust and risk perception (77). When doctors share information, they improve mutual understanding and foster trust (78, 79). In the meantime, healthcare vulnerabilities, for instance, ailment, injury, and demise, are common; any treatment must account for such unpredictability, and practitioners are encouraged to exercise appropriate approaches, for example, lay terms, to acknowledge uncertainty to mend patients’ risk perception (80, 81). According to research, positive PPC can increase medical treatment satisfaction, which is compatible with the framework described by Ong (82). Prior research has found that trust significantly impacts service quality and patient satisfaction (83, 84).

Physical infrastructure assesses the patient’s perspective of service quality and the clinic’s tangible services. This gage considers the facility’s cleanliness and management and the accessibility of tangible facilities, including patient rooms, technical capability, medical testing rooms, blood banks, patients’ wards, beds, ambulances, waiting areas, and operating rooms. Numerous research has previously been conducted to determine the impact of tangible services on quality delivery (59, 60, 85). Creating a safe atmosphere that greatly aids patients in completing their recovery. Qualified hospital workers must work to enhance the external conditions of the healthcare institution, so an environment would greatly assist patients in recovering on time and maintaining a healthy lifestyle (86, 87). Patient trust and satisfaction are crucial in mediating the association between perceived quality and desire to return to the institution (88). Han, Kim (89) investigated the link between trust, satisfaction, and loyalty in hospitals. He discovered that the association is considerable and fine, and trust is essential as an intervening variable, particularly in healthcare. Numerous studies also utilized trust as a prospective mediating variable in patient satisfaction-related associations, i.e., cultural competence of nurses and PS Tang (90), PS and patient commitment Durmuş and Akbolat (91), and PS and patient loyalty (28). Based on the above evidence, we suppose:

*H3*: Patient trust in general practitioners will mediate the effect of practitioner-patient communication and patient satisfaction.

*H4*: Patient trust in general practitioners will mediate the effect of cleanliness and patient satisfaction.

**Figure 1:**
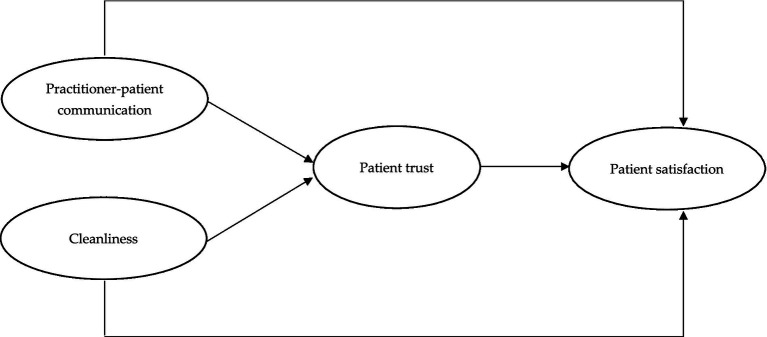
Hypothesized model.

Drawing on a retrospective literature review, the authors developed a hypothesized model, illustrated in Figure 1, to explore how PT mediates the association between practitioner-patient communication, cleanliness, and patient satisfaction.

## Method

### Population and research sample

Using a random sampling technique, the respondents were drawn randomly from the patient visited (Outpatients Department) OPDs of government healthcare institutions in three major Saudi Arabian cities, Riyadh, Jeddah, and Dammam. In this study, participants had access to medical services at the targeted hospitals from March to May 2024. The examination must be finished within 3 months to limit inaccuracies in retroactive reports covering longer times. As a result of the “random sampling” technique, an illustrative sample of the focus group was obtained. Kline (92) suggests that the sampling size in the “Structural Equation Modeling (SEM)” approach should be above 200 respondents to decrease sample error. As a result, this study randomly sampled 600 patients from four government healthcare institutions in three major cities, including Riyadh, Jeddah, and Dammam, spanning the country’s geographical variety. After screening the returned surveys for completion, logical scoring, and conformity to scale, 497 survey responses with an 82.83% response rate were retained for further research.

### Demographic features

Males comprised 61.37% of the population, while females comprised 38.63%. More than 60% of patients are above 40. By and large, In terms of education, around 45.68 had a secondary level education, 37.22% had an undergrad, and master’s degree holders accounted for 17.10%. (see Table 1).

**Table 1:** Demographics details.

Description	No.	Percentage
Gender		
Male	305	61.37
Female	192	38.63
Age		
20–29	53	10.66
30–39	126	25.35
40–49	169	34.01
≥50	149	29.98
Education		
Secondary	227	45.68
Bachelor	185	37.22
Master	85	17.10

### Research procedures

A quantitative survey was utilized to assess the associations among the research’s suggested theoretical models, practitioner-patient communication, and cleanliness on patient satisfaction in three major cities of the Kingdom, with a mediating influence of patient trust. The cross-sectional approach needed data to be acquired from a population sample. The data was collected from the participants using a self-administered structured questionnaire. Two data types were gathered to evaluate the study hypotheses based on the participants’ demographical features and structured responses. Based on the recommendations of Brislin (93), the original questionnaire was translated from English to Arabic before being distributed to respondents. It was discovered that the two translations are extremely similar. The participants were asked to complete a study questionnaire and offer feedback on the inquiry using a “5-point Likert scale ranging from “(1 = strongly disagree and 5 = strongly agree).” Before the study, the researcher informed all respondents of the research’s goal and ensured their privacy.

### Measurement of variables

We adapted this questionnaire from previous studies. “Practitioner-patient communication (PPC)” was evaluated using a four-item scale created by Andaleeb (94). “The practitioners were eager to answer any questions,” and the scale’s reliability was 0.89. “Cleanliness” was measured using the four items defined by Han (95), and Bitner (96). The sample item was “The front side of the hospital is clean,” the scale’s reliability was 0.89. We used a five-item scale to asses PS, created by Dagger (97) to assess PS. A sample question was, “I feel good about coming to this clinic for any treatment,” This scale’s alpha was 0.91. We used four items derived from Koschate-Fischer and Gartner (98) to assess patients’ trust. The sample item was “I expect the clinic to deliver its promise,” the scale’s dependability was 0.89.

### Common method bias (CMB)

Data gathered simultaneously from a singular source may face bias issues that raise significant doubts about the research’s validity. “Harman’s single-factor” test assessed the bias problem (99). The results indicated that every element of the proposed model might be categorized into 4 categories, with the first component accounting for 38.79% of the variation. This number indicates that prevalent biases are under 50%. Therefore, there is no issue of bias in our data.

### Data analysis

We used an SEM technique to evaluate research hypotheses through AMOS 25.0 (100). We utilized Anderson and Gerbing (101) recommendation of a 2-step structural equation modeling approach where we first performed confirmatory factor analysis (CFA) for model appropriateness. After that, a final hypothesized structural model was analyzed to test the relationships among all variables. Several fit indices, metrics such as “*χ*2/df, comparative fit index (CFI), standardized root mean square residual (SRMR), Tucker-Lewis index (TLI), and root mean square error of approximation (RMSEA)” were utilized throughout the conduct of confirmatory factor analysis (CFA). Table 2 presents descriptive data, encompassing relationships among all variables, averages, and standard deviations.

**Table 2:** Descriptive statistics.

Variables	AVE	Mean	SD	Correlations						
				1	2	3	4	5	6	7
1. Age	-	5.65	2.85	-						
2. Gender	-	2.39	1.46	0.9	-					
3. Education	-	3.80	1.89	0.13*	0.06	-				
4. PPC	0.71	2.13	1.35	0.05	0.10	0.19**	-			
5. CL	0.68	1.89	1.06	0.09	0.01	0.07	0.43**	-		
6. PT	0.63	1.40	0.87	0.02	0.04	0.02	0.39**	0.31**	-	
7. PS	0.67	2.98	1.13	0.14*	0.09	0.16**	0.43**	0.42**	0.49**	-

AVE, average variance extracted; PPC, practitioner-patient communication; CL, cleanliness; PT, patient trust; PS, patient satisfaction. *p < 0.05, **p < 0.01.

### Descriptive statistics

Table 2 presents the statistical metrics for each measured variable’s mean, “standard deviation (SD),” AVE, and correlations. The mean values ranged from 1.40 to 5.65, whereas the SDs varied from 0.87 to 2.85. Table 2 indicates that the correlations in all examined variables are positive and substantial. Table two further demonstrates the “discriminant validity” of each component since the empirical values of “average variance extracted (AVE)” exceed the inter-correlational values, with AVE values also surpassing 0.5 (102).

### Measurement model

This study evaluated the measuring model utilizing “Confirmatory Factor Analysis (CFA)” (92, 103); the standard factor loadings, Cronbach’s alpha, and composite reliability (CR) for each component are presented in Table 3. The alpha coefficients for Practitioner-Patient Communication, Cleanliness, Patient Trust, and Patient Satisfaction are 0.92, 0.91, 0.90, and 0.88, respectively. These alphas are above the suggested threshold of 0.70 (104, 105). The standardized factor loadings for Practitioner-Patient Communication varied from 0.78 to 0.85, 0.71 to 0.84 for Cleanliness, 0.71 to 0.88 for Patient trust, 0.70 to 0.82, and 0.71 to 0.81 for Patient satisfaction. All loadings of factor are more than 0.50 (104). The “composite reliability (CR)” ranged from 0.87 to 0.91 for Practitioner-Patient Communication, Cleanliness, Patient trust, and Patient satisfaction, which is above the recommended value of 0.60 (106).

**Table 3:** Measurement model.

Factor	Items	Loadings	S.E.	*T*	C.R.	Α
PPC	PPC1	0.793	-	-	0.91	0.92
	PPC2	0.788	0.060	13.133**		
	PPC3	0.811	0.064	12.672**		
	PPC4	0.850	0.069	12.319**		
CL	CL1	0.799	-	-	0.87	0.91
	CL2	0.716	0.061	11.738**		
	CL3	0.803	0.062	12.952**		
	CL4	0.846	0.061	13.869**		
PT	PT1	0.823	-	-	0.89	0.90
	PT2	0.789	0.065	12.138**		
	PT3	0.710	0.061	11.639**		
	PT4	0.703	0.061	11.525**		
PS	PS1	0.759	-	-	0.89	0.88
	PS2	0.812	0.063	12.889**		
	PS3	0.763	0.064	11.922**		
	PS4	0.717	0.065	11.031**		
	PS5	0.806	0.065	12.400**		

PPC, practitioner-patient communication; CL, cleanliness; PT, patient trust; PS, patient satisfaction; CR, composite reliability. **p < 0.01.

Additionally, we conducted a serial-wise confirmatory factor analysis to verify that the model accurately represented various components. The proposed four-factor assessment model (Practitioner-patient communication, cleanliness, patient trust, and patient satisfaction) demonstrated an adequate fit to the data: *χ*2 = 2691.56, Df = 948, *χ*2 /df = 2.83, CFI = 0.93, TLI = 0.93, RMSEA = 0.05, and SRMR = 0.04 (Table 4). The proposed four-factor measurement model is the most suitable model among the others presented in Table 4. Every observed item demonstrated significant loadings on its respective latent variables (Table 3). The proposed four-factor model was evaluated against alternative CFA models. The fit indices in Table 4 demonstrate the “components’ convergent and discriminant validity,” providing a robust basis for assessing the proposed 4-factor model.

**Table 4:** CFA results.

Model	*χ* ^2^	Df	*χ*^2^ /df	CFI	TLI	RMSEA	SRMR
4-factor model (hypothesized model)	2691.56	948	2.839	0.93	0.93	0.05	0.04
3-factor model (PPC & CL combined)	7732.46	957	8.080	0.79	0.80	0.13	0.10
2-factor model (PPC, CL & PT combined)	7308.98	957	7.637	0.81	0.81	0.12	0.09
1-factor model	8679.31	963	9.013	0.74	0.74	0.17	0.15

### Hypothesis testing

We employed a comprehensive structural equation model with maximum likelihood estimation in “AMOS” software to assess the hypotheses of our investigation. Simultaneously, the correlations presented in Table 1 and the structural equation modeling findings validated hypotheses 1–2, as illustrated in Table 5. Hypothesis 1 suggests a significant positive correlation between practitioner-patient communication and patient satisfaction. Evidence supporting Hypothesis 1 was identified (standardized *β* = 0.43, *t* = 7.30, *p* < 0.01), as seen in Tables 1, 5. The second hypothesis predicts a favorable correlation between cleanliness and patient happiness. Hypothesis 2 received support (standardized *β* = 0.31, *t* = 4.92, *p* < 0.01).

**Table 5:** Direct effects.

Relationships	*Β*	S.E.	*t*	95% CI
PPC → PS	0.431	0.059	7.305	(0.381, 0.514)
CL → PS	0.310	0.063	4.921	(0.209, 0.471)
PPC → PT	0.356	0.062	5.742	(0.312, 0.601)
CL → PT	0.389	0.059	6.593	(0.219, 0.411)
PT → PS	0.417	0.060	6.950	(0.398, 0.586)

Hypothesis 3 of our study indicates that ‘patient trust will considerably influence the relationship between practitioner-patient communication and patient satisfaction.’ Table 6 illustrates that the *β* coefficient for PPC and PS becomes insignificant (*β* = 0.040; S.E. = 0.059; *t* = 0.678; CI = −0.061, 1.012) when PT is accounted for, however, the indirect beta coefficient is substantial (*β* = 0.148; S.E. = 0.062; *t* = 2.387; CI = 0.337, 0.589). The data indicate that PT mediates the link between PPC and PS. Hypothesis 4 suggests that PT mediates the link between CL and PS. Table 6 indicates that the value of the beta coefficient is considerable, demonstrating a notable mediational mechanism. The indirect link for hypothesis 4 is significant (*β* = 0.162; S.E. = 0.061; *t* = 2.656; CI = 0.259, 0.352). The correlation between CL and PS is rendered insignificant (*β* = 0.009; S.E. = 0.061; *t* = 0.148; CI = −0.001, 0.013).

**Table 6:** Bootstrapping indirect effects.

Relationships	*β*	S.E.	*t*	95% (CI)	
				Lower limit	Upper limit
PPC → PS	0.040	0.059	0.678	−0.061	1.012
CL → PS	0.009	0.061	0.148	−0.001	0.013
PPC → PT → PS	0.148	0.062	2.387	0.337	0.589
CL → PT → PS	0.162	0.061	2.656	0.259	0.352

## Discussion

The primary goal of this research was to measure patient satisfaction with patient-practitioner communication and health care quality, such as cleanliness, using patient trust as a mediating variable. This research was carried out at Saudi government hospitals. Two predictors were used to evaluate patient satisfaction: patient-practitioner communication and health service, e.g., cleanliness. Moreover, the research examined patient trust as a mediator in the association between patient-practitioner communication and healthcare outcomes such as cleanliness and patient satisfaction. This research adds to the body of knowledge and contribution to the healthcare field. While reviewing the literature, it became clear that most research was done in emerging and advanced nations (107, 108). This research concentrates on patient-practitioner communication and the influence of cleanliness on patient satisfaction. It is unique and novel in that it examines the function of patient trust as a mediator in developing nations such as Saudi Arabia. Our findings also support earlier research; all assumptions developed concerning theoretical associations between variables were confirmed in this research. The hypothesis testing results suggest that PPC significantly affects PS, confirming Szasz and Hollender’s study conclusions that PPC is essential to improve patient satisfaction and confidence. The idea that cleanliness benefits patient happiness is consistent with Akmaz and Cadirci (63), who discovered that cleanliness is essential for increasing PS in healthcare. The notion that patient trust positively influences patient satisfaction aligns with the research findings (28). The hypothesis that PPC and cleanliness positively influence patient happiness and that PT mediates this link is also in line with the findings of Liu (28), who investigated general populace satisfaction, loyalty dimensions, and public trust practices. However, no one has specifically investigated the direct influence of PPC and cleanliness on PS with a mediating role of PT in Saudi Arabia’s healthcare industry. According to the findings of this study, patient trust enhances and positively mediates the association between PPC, cleanliness, and patient satisfaction. All hypothesis testing indicates that patient trust mediates the association between PPC, cleanliness, and patient satisfaction. However, no one has specifically investigated the direct influence of PPC and cleanliness on PS with a mediating role of PT in Saudi Arabia’s healthcare industry. Hence, the present study assessed this gap and established that patient trust enhances and positively mediates the association between PPC, cleanliness, and patient satisfaction. Moreover, the findings of the mediation analysis show that the supposed hypotheses are wholly acceptable. Hence, the present study assessed this gap and established that patient trust enhances and positively mediates the association between PPC, cleanliness, and PS. Moreover, the findings of the mediation analysis show that the supposed hypotheses are wholly acceptable.

### Practical recommendations

This research gives various recommendations for the hospital to boost patient satisfaction. First, it is suggested that hospitals offer interpretation services. The findings of this study complement those of recent research Pinto Taylor (109) on the influence of medical interpretation services on healthcare quality. Secondly, the hospital must manage the physician’s workload effectively. This guideline pertains to the duration of engagement with the patient, as previously discussed. The issue of physician workload or staffing levels leads to diminished time and energy devoted to patients, thus impacting the quality of patient-physician communication. Hospital performance enhancement is frequently realized by prioritizing effectiveness and efficiency while augmenting physicians’ workloads. Nonetheless, efficacy and efficiency may adversely affect patients’ perceptions of the quality of communication with their physicians. Patient comfort and physician workloads are critical factors for hospitals aiming for operational efficiency. Government hospitals must provide a relaxing, clean, and comfortable environment in OPDs (Outpatients Departments). Clinical waste must be handled properly to avoid an unpleasant smell in OPD’s washrooms. The pleasant and clean environment of hospital OPD’s seating areas and doctor’s checkup rooms gave an impression of serenity and contentment. Patient satisfaction strongly relies on the practitioner’s ability to communicate and connect with patients. That is why proper practitioner communication and ethical and behavioral training in exchanging pleasantries and expressing empathy and kindness are necessary. To align services with patient expectations, practitioners’ tolerance for interacting with illiterate and impoverished patients should be improved by acquiring expression control skills. Moreover, advanced training sessions should be organized to equip practitioners to address the needs of patients from the lowest socioeconomic strata, as Saudi Arabia is home to millions of poor workers from different countries. It is essential to provide ethical and competent service delivery to enhance the quality of practitioner-patient contact in no-cost public outpatient clinics in Saudi Arabia. Launching patient satisfaction surveys helps improve the quality of medical care service delivery. Practitioners must have excellent medical expertise and the ability to transmit knowledge efficiently and sympathetically. Understanding the determinants of patient satisfaction may assist physicians, hospitals, and policymakers in devising and executing effective strategies to enhance healthcare services. This study indicates that patient satisfaction with practitioner communication is influenced by enhancements in the physician’s demeanor and organizational variables contributing to elevated patient satisfaction. Comprehensive patient satisfaction models can assist policymakers in identifying patient needs, defining practitioner and patient responsibilities, managing demand and capacity, and ensuring the requisite quality of services.

### Limitations and scope for future studies

This study has specific limitations. The present study employed quantitative approaches; future studies may apply qualitative or mixed procedures to provide more persuasive outcomes. The patient’s assessment of healthcare services is a distinctive method, and employing a quantitative approach such as a survey may not accurately reflect each patient’s rating. In the next investigation, using qualitative methods instead of quantitative procedures may yield a more profound comprehension of the relationship among PPC, cleanliness, PT, and PS. The findings of this study were obtained from research carried out in three cities. The privately owned institutions’ healthcare services differ from those offered by public hospitals in Riyadh, Jeddah, and Dammam. It is advisable to conduct further research in private healthcare facilities to enhance understanding of the relationship between both variables in the presence of an intervening variable. Thirdly, we conducted our inquiry in three cities because of time limitations. This study might be extended to more cities or countries to generalize the findings in the future. Fourthly, we acquired data from outpatient departments; thereafter, data from discharged patients must be obtained to evaluate the study model’s relevance to this demographic. Data obtained from patients may be susceptible to bias. Participants may not receive precise information on service quality, and their responses may change if they take the survey at a clinic. Fifth, participants may not have provided accurate information on service quality, although their responses could have varied had they completed the survey in the clinics. This study used a cross-sectional approach; future studies could opt for other research methods. Finally, the current study included a mediating effect. Future studies may focus on utilizing hospitalized and outpatients as moderating factors to identify differences in medical institutions.

## Conclusion

In conclusion, this study significantly contributes to the literature on patient satisfaction in the healthcare sector. It presents quantitative evidence that patient trust is critical in forming patient satisfaction. Trust must be formed to determine patient satisfaction. Although practitioner-patient communication and cleanliness are important predictors of patient satisfaction, patient trust acts as a mediator between practitioner-patient communication, cleanliness, and patient satisfaction, and patient trust is significantly connected to practitioner-patient communication and PS. Moreover, healthcare organizations have to assess PS with services, the degree of confidence in themselves and healthcare staff, and their level of dedication. Assessments may assist healthcare organizations in identifying appropriate solutions. These findings have practical implications for Saudi Arabian healthcare officials and practitioners, who must emphasize hospital quality and services and gain from marketing theory to form successful client relations.

## Data Availability

The raw data supporting the conclusions of this article will be made available by the authors, without undue reservation.

## Appendix

**Table:**

Practitioner-patient communication (PPC)
The practitioners were willing to answer any questions
I received adequate explanation of any tests I had to undergo
I was given adequate information on my treatment
I was given adequate information on my health condition
Patient satisfaction (PS)
I feel good about coming to this clinic for my treatment
My feelings towards the clinic are very positive
Overall, I am satisfied with the clinic and the service it provides
I feel satisfied that the results of my treatment are the best that can be achieved
The extent to which my treatment has produced the best possible outcome is satisfying
Patient trust (PT)
I expect the clinic to deliver its promise
I trust hospital
I rely on hospital
Hospital is safe
Cleanliness (CL)
The front side of the hospital is clean
There were adequate numbers of bathrooms and toilets in the ward
Toilets were kept clean
The hospital was always neat and clean
